# What information do patients want across their cancer journeys? A network analysis of cancer patients’ information needs

**DOI:** 10.1002/cam4.1915

**Published:** 2018-12-07

**Authors:** Yvonne Tran, Klay Lamprell, Brona Nic Giolla Easpaig, Gaston Arnolda, Jeffrey Braithwaite

**Affiliations:** ^1^ Centre for Healthcare Resilience and Implementation Science, Australian Institute of Health Innovation Macquarie University North Ryde New South Wales Australia

**Keywords:** cancer journey, information needs, network analysis, patient experience, patient satisfaction

## Abstract

**Background:**

Patients want information across their cancer journeys. We investigated what sort of information they want and at what stage in the cancer journey by examining English patients’ satisfaction derived from ratings of their care.

**Methods:**

Using patient experience data from 209 Clinical Commission Groups (CCGs) involving 72 788 respondents in 2016, overall patient satisfaction ratings and information needs questions were extracted. Novel network analysis techniques were used to construct an undirected weighted concentration network to assess the relationship between information needs and patient satisfaction.

**Results:**

From the network analysis, we found that patients value information early in the pathway; there were higher associations between patient satisfaction and when information needs are met in earlier phases of the cancer journey. Across the stages of the cancer journey, strong associations between information needs and patient satisfaction emerged during diagnostic testing and also at those points when patients received information provided by the clinical nurse specialists. A mixture of strong and weak associations between patient satisfaction and information needs was found during later phases of the cancer journey, specifically when patients move from treatment to home care. Our study identified that meeting needs for information related to supportive care may be a weaker factor in patient satisfaction than meeting needs for information related to the patient's disease, its treatment and the side effects of treatment.

**Conclusion:**

If patients have their information needs met, especially during stages within the cancer journey when information needs are highest, they are more likely to be satisfied with their care. Our study has implications for information giving and improving patient satisfaction in cancer care.

## 1 INTRODUCTION

A diagnosis of cancer leads to a significant level of adjustment for patients and can seriously affect quality of life.^1^, ^2^, ^3^ We know that it is common for cancer patients to experience uncertainty about their prognosis, a reduced sense of control over their lives, unpleasant and debilitating side effects from treatment, increased dependence on others, and disruptions to family, work, and social life.^4^, ^5^, ^6^ Addressing information needs at different stages of their cancer journey can help patients cope with the challenges of a cancer diagnosis by facilitating a sense of control, counteracting feelings of helplessness.^7^ Adequately informed cancer patients have been reported to experience lower levels of anxiety and depression and report a better quality of life.^8^ Information provision can improve treatment compliance, create more realistic expectations, promote participation and self‐care, and generate feelings of safety and security.^9^, ^10^ The benefits of information provision in the diagnostic and treatment phases of cancer care may reduce the need for supportive care in survivorship.^8^, ^11^

The response to information needs is also playing an increasingly important role in patients’ satisfaction with care. There is evidence of the links between information provision and patient perceptions of quality of care.^12^ Patients’ perceptions are increasingly used as the metric for determining high quality of care as well as a means of assessing health care delivery in hospitals and health services.^13^ But we do not know enough about a crucial question: if information needs are met across different stages of the care journey, do patients rate their satisfaction with care more highly? A related key question is as follows: at what stages in the cancer journey is information most valued by patients?

Broadly, the cancer journey starts with a Diagnostic test and then moves through Finding out what was wrong, to Deciding best treatment, to Operation, to Hospital care inpatient, to Hospital care outpatient, and to Home care support.^14^ The aim of this paper is to examine the relationship between receiving information throughout a cancer journey and a patient's overall rating of satisfaction with their care. The dynamics between the information given and received, and overall satisfaction with care, are examined using an emerging network analytic method pioneered in clinical psychology and psychopathology studies, referred to as a psychological network.^15^ It is applied for the first time to aggregated data drawn from extensive questionnaires of cancer patients’ experiences with their care. The approach is ultimately based on graph theory, where network approaches have been used to describe social relations, biological structures, and information networks.^16^, ^17^

In our study, we apply conventional statistical methods to the novel analysis of a psychological network. The nodes from the network represent the observed variables of patient experience at different points of the cancer journey. The links between nodes (“edges”) show the strength of relationships, calculated using the concentration of partial correlation. In all methods, networks consist of nodes from observed variables, but psychological networks differ from other methods in that edges are not observed but estimated.^18^ In psychological networks, the edges represent statistical relationships; for example, in concentration networks they are a network of partial correlation coefficients. Concentration networks can be used to model unique interactions, map out multicollinearity and predictive mediation, be indicative of potential causal pathways, and highlight latent variables through clustering of nodes.^19^

## 2 METHOD

### 2.1 Data

We analyzed publicly available anonymous data from the 2016 National Cancer Patient Experience survey (NCPES) commissioned by the National Health Service (NHS) England. The NCPES was conducted by post and sent out to all English cancer patients aged 16 years and over. Participants had a primary diagnosis of cancer and were admitted to an acute or specialist NHS Hospital in England that provided adult cancer services as inpatients or day cases. Patients were discharged within a specified three‐month sampling period; for the 2016 NCPES, this period comprised discharges between 1 April 2016 and 30 June 2016. There were 72 788 respondents, with a response rate of 66%, with results summarized for each of 209 NHS Clinical Commission Groups (CCGs), geographical entities covering the country. CCGs are clinically led groups set up to organize the delivery of NHS services. Within each CCG, there are district health care services such as primary care, hospital care, community care, and mental health. Inclusion and exclusion criteria for the survey can be found in the NCPES, 2016 guidance manual.^14^

### 2.2 Information needs questions

The NCPES collected patient experience information from patients who responded to questions mapped to a structured cancer journey. The survey begins with questions about respondents’ initial GP visit prior to their cancer diagnosis, follows with questions about diagnosis and treatment, and proceeds with questions throughout the management of their cancer.^14^ There were 53 questions reported as the percentage of patients who recorded a positive response, for instance “Yes, completely.” The scoring key for positive responses is found in the technical documents from the NCPES. Patients reporting a neutral response such as “I Don't Know” were excluded from the denominator for these calculations.^14^ From the positive performance indicators, 23 items were identified as information needs questions, that is they were either recognized in the Macmillan guide^20^ as information needs questions, or they were questions relating to information needs as defined by the NHS statutory guidance for CCGs^21^ (Table 1 for the 23 chosen questions). For the information needs questions, the percentages reported for each CCG were interpreted as the proportion of the CCG where information needs were satisfied.

**Table 1:** Descriptive statistics for information needs questions from the NCPES survey in 209 CCGs

Information needs Questions	Mean (%)	SD (%)	Minimum (%)	Maximum (%)
Diagnostic tests				
Q5: Beforehand, did you have all the information you needed about your test?	93.9	2.11	84.4	98.2
Q7: Were the results of the tests explained in a way you could understand?	78.4	4.03	59.1	86.9
Finding out what was wrong with you				
Q10: Did you understand the explanation of what was wrong with you?	73.0	3.54	63.5	83.5
Q11: When you were told you had cancer, were you given written information about the type of cancer you had?	72.1	4.51	56.2	84.3
Deciding the best treatment for you				
Q12: Before your cancer treatment started, were your treatment options explained to you?	82.4	3.44	70.8	91.1
Q13: Were the possible side effects of treatment(s) explained in a way you could understand?	72.3	3.57	61.5	81.8
Q14: Were you offered practical advice and support in dealing with the side effects of your treatment(s)	65.2	4.53	48.0	76.8
Q15: Before you started your treatment(s) were you also talk about any side effects of the treatment that could affect you in the future rather than straight away?	54.5	4.26	44.4	71.2
Clinical nurse specialist				
Q17: Were you given the name of a clinical nurse specialist who would support you through your treatment?	90.3	3.24	78.3	96.9
Q19: When you have had important questions to ask your clinical nurse specialist, how often have you got answers you could understand?	87.8	3.47	76.6	94.6
Support for people with cancer				
Q20: Did hospital staff give you information about support or self‐help groups for people with cancer?	83.3	5.18	64.2	94.2
Q21: Did hospital staff discuss with you or give you information about the impact cancer could have on your day to day activities?	80.8	4.15	67.6	90.7
Q22: Did hospital staff give you information about how to get financial help or any benefits you might be entitled to?	55.6	6.65	38.3	81.6
Q23: Did hospital staff tell you that you could get free prescriptions?	80.1	4.62	61.7	91.7
Operations				
Q25: Beforehand, did you have all the information you needed about your operation?	95.7	1.85	90.2	100.0
Q26: After the operation, did a member of staff explain how it had gone in a way you could understand?	78.4	4.09	63.5	90.0
Hospital care as an inpatient				
Q38: Were you given clear written information about what you should or should not do after leaving hospital?	85.4	3.68	74.6	94.1
Q39: Did hospital staff tell you who to contact if you were worried about your condition or treatment after you left hospital?	93.7	2.38	83.0	99.1
Hospital care as an outpatient				
Q44: Beforehand, did you have all the information you needed about your radiotherapy treatment?	86.0	4.98	68.2	97.7
Q45: Once you started your treatment, were you given enough information about whether your radiotherapy was working in a way you could understand?	59.7	7.08	35.7	76.5
Q47: Beforehand, did you have all of the information you needed about your chemotherapy treatment?	83.9	3.94	72.2	94.6
Q48: Once you started your treatment, were you given enough information about whether your chemotherapy was working in a way you could understand?	66.9	5.44	48.5	81.1
Home care and support				
Q49: Did the doctors or nurses give your family or someone close to you all the information they needed to help care for you at home?	57.7	4.29	45.8	69.5

### 2.3 Overall patient rating of care

A global patient rating was captured by the final question: “Overall, how would you rate your care?” Respondents were asked to circle a value from zero to ten on a scale of “very poor” to “very good.” CCG performance was reported as the average rating provided and presented as a percentage. In this study, we interpret this rating as representing the degree of satisfaction with care.

### 2.4 Data analysis

#### 2.4.1 Network estimation and visualization

All statistical analyses were conducted using R (RStudio v1.1.442, Boston, MA). Descriptive statistics were first calculated for the 23 information needs questions and the global patient satisfaction index question for the 209 CCGs. To examine the relationships regarding different information needs through the cancer journey, we conducted a network analysis, using the qgraph^22^ and igraph^23^ R packages.

We first computed a correlation matrix of the 23 information needs questions and the global patient satisfaction rating from all 209 CCGs. An undirected weighted concentration network was constructed based on the correlation matrix. Connections between the 23 questions and the global rating (“edges”) depict partial correlations between each pair of nodes after controlling statistically for all other variables. The R package qgraph automatically implements a graphical regularization in combination with an extended Bayesian information criterion (EBIC) model selection.^22^ In this method, 100 different network models were estimated with different degrees of sparsity and the model with the lowest EBIC is selected. The network is estimated using a graphical Gaussian model with least absolute shrinkage operator regularization. This method computes regularized partial correlations between pairs of nodes, eliminating spurious connections from the influence of other nodes within the network, and shrinks trivial and small associations to zero.^24^

The nodes in the graphical representation of the network represented the scores from the CCG for the 23 information needs questions at different points of the cancer journey and the overall patient satisfaction rating. The edges show the strength of relationship between the nodes, with thicker lines representing stronger relationships. Blue lines represent a positive relationship, and red lines represent a negative relationship. Within the network, nodes are positioned to visually represent the relative strength of their connections; nodes that are more strongly connected are depicted closer together and nodes nearer to the center of the graph have the strongest connection to other nodes.^24^

Other network metrics were generated. To quantify the importance of each node in the network, centrality indices were computed. Centrality indices indicate how connected and relevant a node is within the network and identifies the most influential nodes. Three centrality indices were examined: “betweenness” measures the number of times a given node lies in (acts as a bridge to) the shortest path length between any other pair of nodes; “closeness” measures the average distance of a node from all other nodes in the network; and “strength” measures the sum of the edge weights attached to each node (or the number of connections).^24^ For each of the centrality indices, higher values reflected greater centrality in the network. The scores reported were standardized to z‐scores. Network density was also calculated; this is a measure of the percentage of connections over the total number of possible connections.^22^ To examine whether the nodes acted as a single system or in smaller clusters of nodes, community detection was also implemented, using the spin glass algorithm from the R package igraph.^23^

## 3 RESULTS

The mean score for patient satisfaction rating of care was 87.1% (SD = 1.69%; range 82.0%‐90.4%). Table 1 presents descriptive statistics for the 23 information needs question from the 209 CCGs. Information needs questions that attracted a greater than 90% positive score indicating greater information needs are met, covered the span of the cancer journey from diagnosis (Q5), through hospital stay (Q17, Q25) to after leaving hospital (Q39). Low positive responses (<60%) were found in questions regarding information about side effects in the future (Q15), possible financial assistance (Q22), radiotherapy (Q45), and information for carers after leaving hospital (Q49).

**Figure 1:**
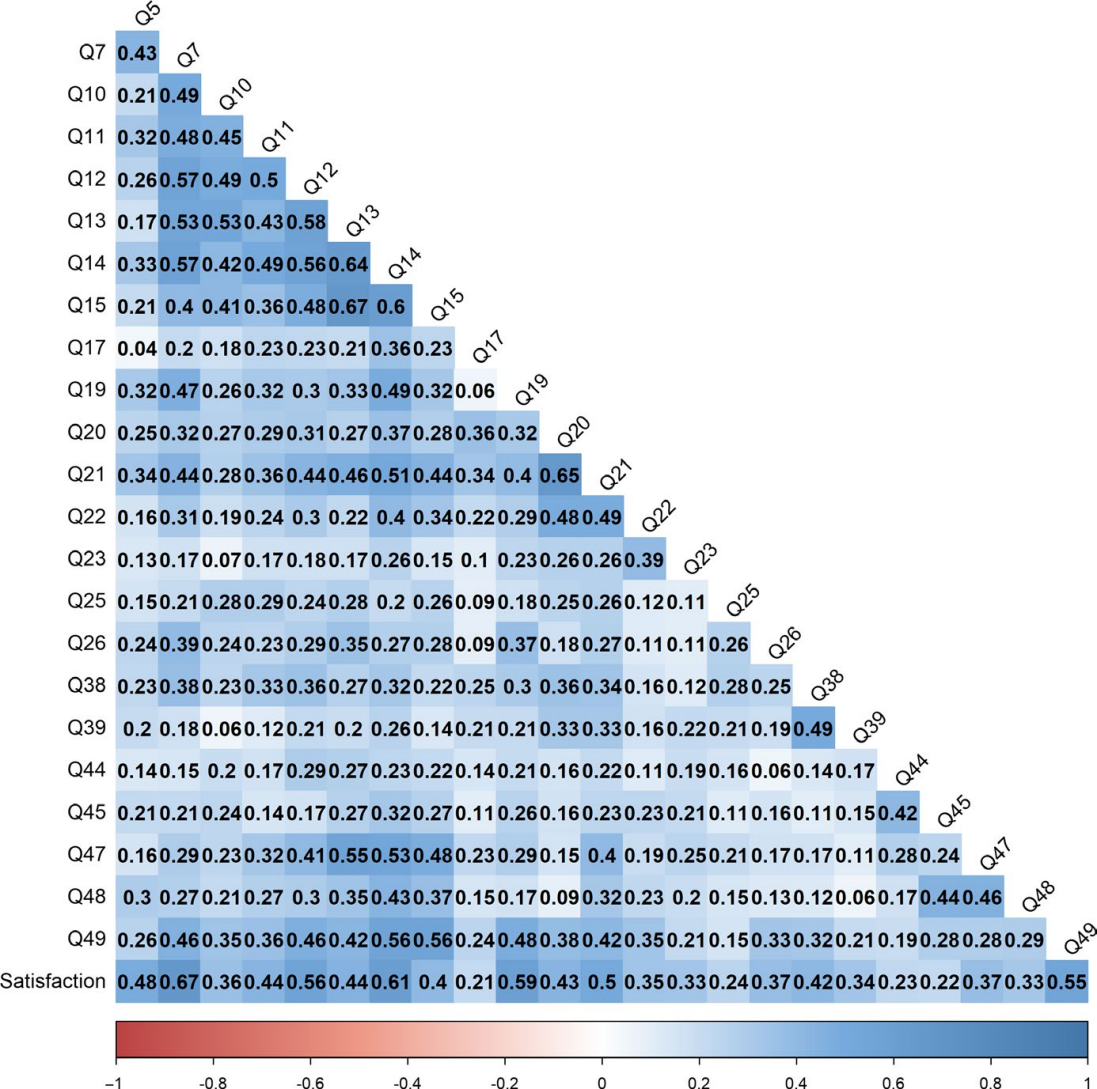
Correlation matrix of information needs questions and patient satisfaction. Blue, positive correlations; Red, negative correlations

Figure 1 shows the 24 × 24 correlation matrix of information needs questions and patient satisfaction used to generate the network structure. All correlations observed in this matrix were positive, with the depth of blue coloring indicating the relative strength. Strong correlations can be seen between information needs questions during the early journey phases and with patient satisfaction rating.

**Figure 2:**
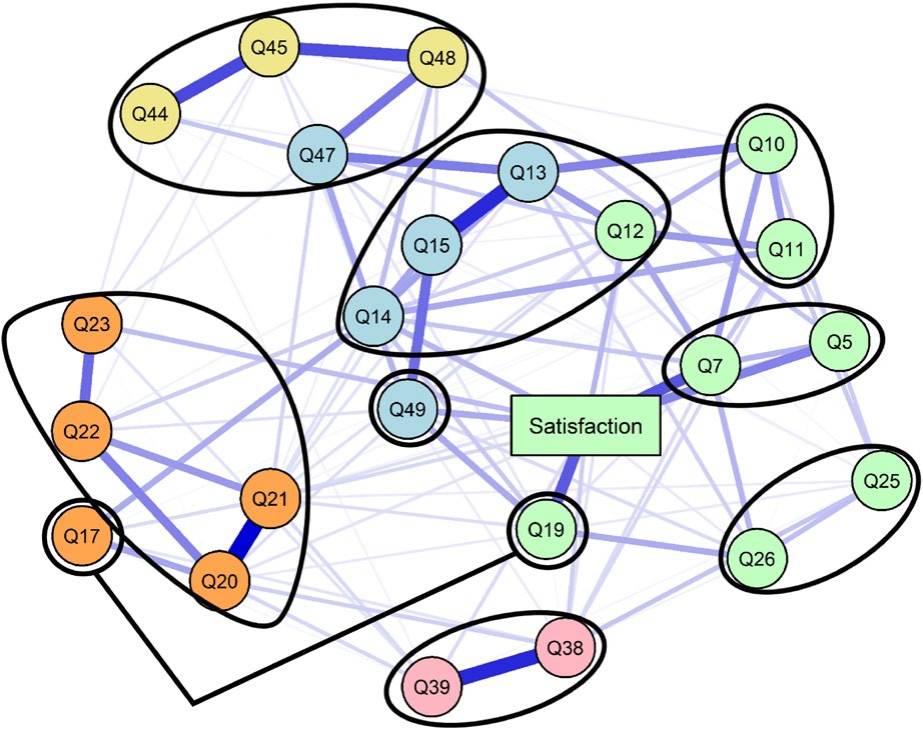
Partial correlation network depicting information needs with overall patient satisfaction with care. The nodes represent the 23 (circle) information needs questions with one overall satisfaction node (rectangle). The edges reflect the magnitude of the association between the nodes with thicker edges representing stronger relationships. It is color coded to the information communities identified using the spin glass algorithm in the network analysis. In black circles are the different questions from the parts of the cancer journey identified in the NCPES

Figure 2 shows the resultant network structure of global patient satisfaction (rectangular node “Satisfaction”) with information needs (all other circular nodes). There were 119 connections between the nodes, resulting in an edge density of 43.1%. There were only positive associations. From the network diagram, several nodes are closely and strongly connected to each other as indicated by their proximity and strength (thickness of the connecting edges). Q20‐Q23 are information needs questions relating to support for people with cancer and are closely connected, with strongest connection between Q20 (support or self‐help group) and Q21 (impact on day to day activities); *r* (partial correlation) = 0.47. Questions Q38 and Q39, relating to support after leaving hospital are strongly connected (*r* = 0.39). Information about radiotherapy and chemotherapy (Q44, Q45, Q47, and Q48) are also closely connected (eg, *r* = 0.38 for Q44 and Q45). Strong connections were also observed between Q13 and Q15 (both relating to side effects) and Q49 (care at home); for example, *r* = 0.33 between Q15 and Q49. The nodes most strongly associated with overall satisfaction with care comprised of Q5 and Q7, which relate to information provided during diagnosis (*r* = 0.20 and 0.31), and Q19 regarding understanding information provided by a clinical nurse specialist (*r* = 0.28).

Network community detection identified five clusters for the network structure indicating the network consists of defined clusters as opposed to a single system. Each cluster is represented by a different node color in Figure 2. The network diagram also identifies, grouped by black circles, the sets of questions from each stage of the cancer journey as described in the NCPES. There were close overlaps found between the community detection clusters and the cancer journey groups of information needs questions. The yellow‐colored cluster (Q44, Q45 and Q48) comprised information needs questions associated with radiotherapy and chemotherapy. Circled in black are four nodes which also included Q47; these are all questions from the outpatient care of the cancer journey. Question 47 was found to group with the side effects (Q13‐Q15) and home care (Q49) in the network community cluster (blue‐colored). The blue‐colored cluster comprised questions from three different NCPES cancer journey stages, with potential side effects surrounding treatment and care being the common theme. The orange‐colored group consisted of information needs questions for support during cancer treatment (Q20‐Q23) and included node Q17, which related to knowing the name of the clinical nurse specialist. Q38 and Q39 were associated with the leaving hospital stage of the cancer journey and were found to be in a group of their own (pink‐colored).

The largest group (green‐colored) contained the information needs questions that were found to cluster with the rating of overall satisfaction with care. Of these, Q5 and Q7 were questions relating to the “diagnostic tests,” Q10, Q11, and Q12 related to “finding out what was wrong” and “best treatment options.” Q25 and Q26 related to operations and Q19 were a question regarding clarity of information provided by the clinical nurse specialist. All the questions clustered together with the global satisfaction rating comprised information needs questions from an earlier phase of the cancer journey up to and including the operation phase.

**Figure 3:**
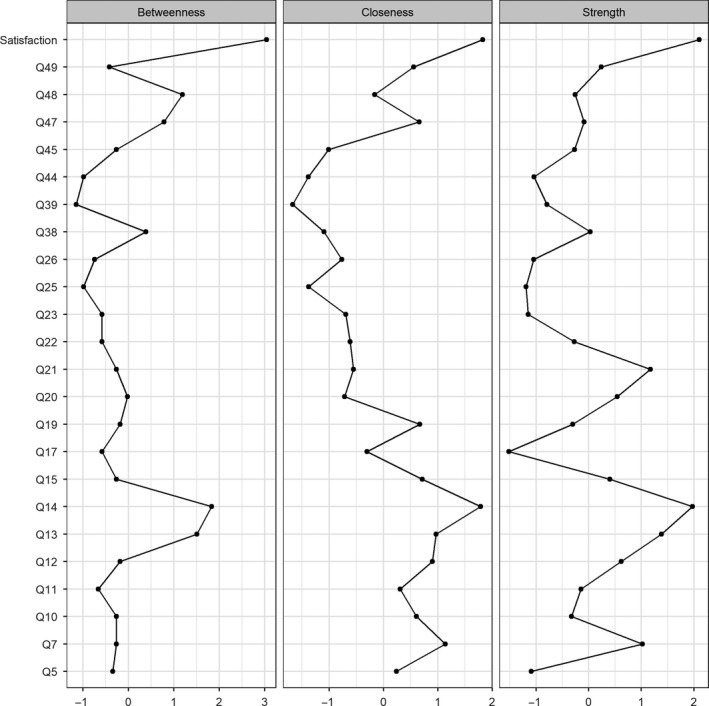
Centrality plot for the concentration network depicting the betweenness, closeness, and strength at each node

Figure 3 shows the result of the centrality analyses with betweenness, closeness, and strength indices. Node centrality analyses identified global satisfaction with care as the node exerting the strongest influence within the entire network. Node Q14 (“Were you offered practical advice and support in dealing with the side effects of your treatment(s)”) also possessed strong centrality characteristics. This was indicated by these two nodes having the highest number of connections (strength), meaning they are related to the other information needs nodes directly. Global satisfaction had the highest “betweenness” score, which showed that it is the node that acts as a bridge to the shortest path between any other pair of nodes the greatest number of times. Global satisfaction also had the highest “closeness” score, which means it travelled the shortest distance to all the other nodes, compared to the other nodes within the network. Similarly, Q14 was also central in this network, with high “betweenness” and “closeness” scores. Information about the impact of cancer on day to day activities (Q21) also had a high number of connections (“strength”) but with a weaker association and was not as influential as global satisfaction and Q14, as it had lower “betweenness” and “closeness” scores.

**Figure 4:**
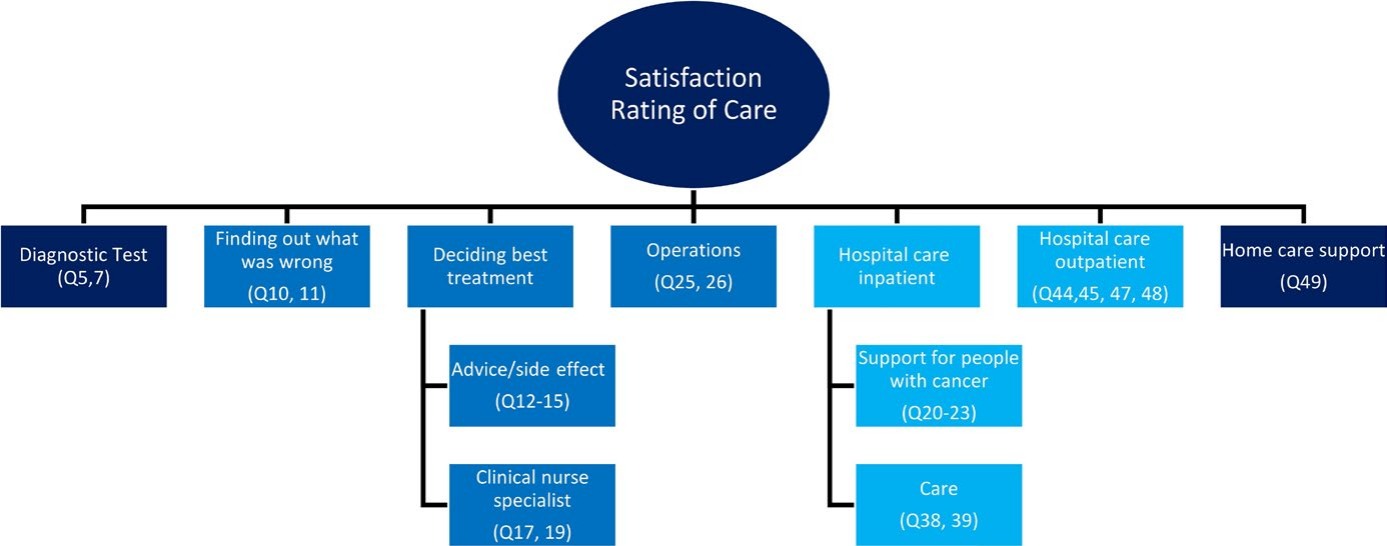
Schematic representation of the associations between the information needs nodes and global patient satisfaction with care over the cancer journey. Strong associations are depicted in darker blue moderate/fluctuating associations are depicted blue weak association are in light blue

Figure 4 shows a schematic summary of the relationships between global satisfaction with care and when information needs are encountered through the cancer journey. This is a representative interpretation of the findings from the network analysis combining the strengths of the connections, proximity of the nodes, community clustering, and centrality indices. Strong, moderate, and weak associations are represented as gradients of blue with strong associations in a darker shade of blue and weaker associations in a lighter shade of blue. Strong associations were found between nodes at the start of the journey with information regarding diagnostic tests and with the node relating to home care support. Associations with global satisfaction fluctuated in the middle phases of the cancer journey with a mixture of weak and strong associations during the phase of finding out what is wrong and up to the operation stages of the journey; this was represented as a moderate association. The associations that were weakest or furthest away for the information nodes occur during deciding hospital care inpatient and outpatient, represented in light blue.

## 4 DISCUSSION

Most cancer patients wish to be well informed at all stages of their cancer journeys.^25^ Patients want information about their care, and receiving valued information is very strongly related to satisfaction with that care. Information‐seeking behavior continues from diagnosis to follow‐up. Applying a novel network analytic approach to a large database of patients’ views of their care allowed us to examine the interplay and pathways between global satisfaction ratings of care and the meeting of specifically timed information needs through the cancer journey.

Overall, the edges in the network indicated the strength of the positive associations between information needs and satisfaction showing that when higher proportions of patients reported their information needs were met, their global satisfaction rating was also higher. Having greater satisfaction with cancer care has important implications for improving health‐related quality of life and self‐efficacy.^26^ The global patient satisfaction node was found to be the most influential node in this network, with the strongest centrality characteristics, and most predictive capacity. Generally, centrality characteristics are interpreted as identifying the nodes that strongly incite multiple other nodes when activated^27^; in the current study, the results are best interpreted as indicating that satisfaction is a central product of meeting the full range of information needs.

The network analysis highlighted latent groups through community detection. Five clusters were identified. The clusters related to temporal traits of a cancer journey and also to latent themes. For instance, the green‐colored cluster contained information needs questions from earlier phases of the cancer journey, and the blue‐colored group related to information needs associated with side effects. Satisfaction with care was found to cluster with information needs questions associated with earlier phases of the cancer journey. This indicated that meeting information needs encountered up to and including the hospital stay of the cancer journey, before outpatient care, is most associated with satisfaction. Higher levels of need for information have previously been found in other studies to occur during the early stages of the cancer journey such as during the diagnosis of cancer.^12^, ^28^ This has important implications; if a higher proportion of information needs are met in a CCG during this early phase, there is also higher global satisfaction in care from the CCG, bolstering the proposition that providing information at an early stage may lead to fewer supportive needs at later stages of the cancer journey.^11^

In this study, clinical nurse specialists occupied important roles for patients. Being able to understand responses to information requests made to a clinical nurse specialist was strongly related and in close proximity to the global satisfaction node in the network. In the UK, clinical nurse specialists working in long‐term cancer care are key staff dedicated to the care of patients. They are often the main point of contact for patients and their family. However, the provision of clinical nurse specialist care is uneven and can depend on factors such as geographical location or disease grouping.^29^ One major aspect of their role is to insure that the patient knows who to access for information and advice.^21^ Other research has found that patients considered specialist breast care nurses to be a better source of information than other staff,^30^ underlining the importance of the clinical nurse specialist in the role of fulfilling information needs for people with cancer. Strengthening access to nurse specialists may resolve some of the shortfalls of unmet information needs.^9^

Weaker associations with global satisfaction were found for the meeting of information needs regarding support for people with cancer, such as information for self‐help groups, or financial benefits and prescriptions. When considering types of information needs, research has shown that a large majority of patients want information on the specific type of cancer, treatment options, and possible side effects.^31^ In another study, it was found that fewer than 10% of patients sought out or valued information regarding support.^32^ This is substantiated in the network analysis with centrality results demonstrating that “support for people with cancer” nodes were lower in “closeness” and “betweenness,” making these nodes less central and less likely to directly influence this network.

Information needs during outpatient treatment, such as radiotherapy and chemotherapy, also had a weaker association with global satisfaction levels. A qualitative study using in‐depth interviews in an outpatient oncology setting found that although patients want information about diagnosis and treatment, not all patients want further information at all stages of their cancer journey.^33^ This is supported by other studies examining fluctuations in information needs throughout the cancer journey. Although information needs are high during diagnosis, they have been found to fluctuate during treatment and to increase once again during follow‐up.^28^ A similar temporal relationship was found between information needs and patient satisfaction through the cancer journey in our network analysis, demonstrating that meeting information needs when the need is greatest will more likely lead to higher overall satisfaction with care.

### 4.1 Limitations

This paper reports results from a study of data aggregated at CCG level, with scores calculated as the number of definite positive responses as a percentage of all nonneutral responses. The use of aggregated survey data implies certain limitations. For instance, the implications of excluding neutral responses are unclear and future research should identify the reasons behind participants’ neutral responses and how their exclusion may influence the relationship between information needs and satisfaction. The data for the network analysis were obtained from patient satisfaction surveys from CCGs in England; hence, the findings may not be generalizable to cancer care settings elsewhere. The scope of the current paper covers information provision only. While we did not explore the other patient experience questions surveyed, it is possible that there are important relationships between these patient experiences and overall patient satisfaction. The rating of overall care was surveyed after the cessation of care and not at each stage of the cancer journey. However, it is important to note that this adds strength to the finding that early journey nodes (such as diagnosis) are important despite the greater elapse of time from when their care was rated.

## 5 CONCLUSIONS

The results demonstrated the dynamics of information needs and showed how information needs are closely related to global satisfaction ratings of care for people with cancer. We conclude from the network relationship that overall patient satisfaction with care is positively associated with meeting information needs, and stronger associations occur during stages in the cancer journey when information needs are high. This suggests that the type of information and timing of information provision can be utilized to improve patient satisfaction with cancer care.

## CONFLICT OF INTEREST

None of the authors have a conflict of interest in this research.
